# Air Rescue for Pediatric Trauma in a Metropolitan Region of Brazil: Profiles, Outcomes, and Overtriage Rates

**DOI:** 10.3389/fped.2022.890405

**Published:** 2022-06-02

**Authors:** Paulo C. M. Colbachini, Fernando A. L. Marson, Andressa O. Peixoto, Luisa Sarti, Andrea M. A. Fraga

**Affiliations:** ^1^Postgraduate Program in Child and Adolescent Health, Department of Pediatrics, University of Campinas, Campinas, Brazil; ^2^Laboratory of Medical Genetics and Human Genetics, Postgraduate Program in Health Sciences, Health Sciences Department, São Francisco University, Bragança Paulista, Brazil; ^3^Faculty of Medical Sciences, Clinical Hospital of University of Campinas, University of Campinas, Campinas, Brazil

**Keywords:** air rescue, helicopter, overtriage, pediatric trauma, prehospital, triage

## Abstract

Besides ensuring a quick response and transport of trauma victims, helicopter support also involves risks to patients and professionals and has higher operational costs. Studying prehospital triage criteria and their relationship with patient overtriage and outcomes is important, particularly in newly established services and in developing countries with limited health budgets. This could help improve the use of the helicopter rescue and provide better management of the costs and risks related to it. The objective of this study was to determine the epidemiologic and severity profiles of pediatric victims of trauma attended by helicopter in a Brazilian Metropolitan Area to evaluate the outcomes and overtriage rates related to pediatric air rescue in the region. We conducted an observational and retrospective study using 49 hospital and prehospital records from victims of trauma aged <18 years old (yo) assisted by helicopter and then transferred to a tertiary University Hospital. Of the 49 patients, 39 (79.6%) individuals were male, and the mean age was 11.3 yo. Vehicular collisions accounted for 15 (30.6%) of the traumas, and traumatic brain injuries occurred in 28 (57.1%) cases. A total of 29 (59.1%) individuals had severe trauma (Injury Severity Score; ISS >15), and 34 (69.4%) required admission to the intensive care unit. Overtriage varied from 18.4 to 40.8% depending on the criteria used for its definition, being more frequent in individuals aged between 1 and 5 yo. Death occurred in 10 (20.4%) patients. On prehospital evaluation, we classified 29/32 (90.6%) patients with severe trauma according to the Pediatric Trauma Score (PTS ≤8) and 18/25 (72%) according to the Revised Trauma Score (RTS ≤11). Of these, 7/29 (24.1%) and 6/18 (33.3%), respectively, presented ISS <15 at in-hospital evaluation. None of the patients with PTS >8 and 3/7 (42.8%) of those with RTS >11 presented ISS >15. In conclusion, air rescue of pediatric trauma victims was used mainly for critically ill individuals, resulting in rates of overtriage compatible with that found in the literature. PTS showed the lowest rates of overtriage within excellent rates of undertriage.

## Introduction

The use of the helicopter for medical support is important in time critical situations, such as trauma. However, besides ensuring the quick arrival of the medical team at the accident site and the quick extraction of the victim, helicopter transport also involves risks to patients and professionals, can overload specific reference centers, and has higher operational costs (1, 2).

The literature has shown efforts to demonstrate the real benefits of helicopter transport of trauma victims and which type of patient (if any) would benefit the most from its use, being international data conflicting independently of patient age (3, 4). In addition, studies showing some evidence of the benefit of air transport in decreasing the mortality rate (3, 5) have difficulties defining which is the decisive factor for this result (3, 5, 6). However, consensus exists regarding the high rates of overtriage present in helicopter rescues (3, 4, 7–10) and the need for better assessment and triage tools to minimize this problem (11, 12).

In Brazilian operational protocols for aeromedical support, we can find some objective indications for the use of the helicopter, such as transport of organs for transplantation; access and evacuation in difficult-to-reach locations; support for multiple victims or absence of advanced ground support nearby. But despite that, in the most frequent situations involved in air rescue, such as primary prehospital care for the severely injured patient, these same protocols bring several indications which are somewhat subjective, such as “great distance” or “critical traffic,” without actually considering the severity of the trauma. We hypothesized that such subjectivity could lead to the overuse of the aircraft, which is particularly problematic when we consider the costs of the helicopter in the context of a public health system and also the risks associated with this type of transport. Thus, the epidemiologic characterization of the patients transported by air and the study of prehospital assessment for severity classification are important to improve the guidelines for the use of the helicopter and better manage the costs and risks related to it.

The objectives of our study were to determine the epidemiologic profiles of pediatric trauma victims attended by helicopter rescue in a Metropolitan Region of São Paulo state to describe their severity profiles, outcomes, and the percentage of overtriage according to different criteria. We also aimed to correlate the trauma scores used at the scene with the presence of over and undertriage, analyzing their capacity to classify the actual severity of the trauma in prehospital evaluation.

## Methods

Prehospital care to trauma victims in the region of the study is provided by ground rescue units from the municipal Mobile Emergency Care Service (SAMU, *Serviço de Atendimento Móvel de Urgência*), from the State Firefighter Department (FD), and by ground and helicopter units of the Urgency Care and Rescue Group (GRAU, *Grupo de Resgate e Atendimento às Urgências*), of São Paulo Military Police. There are three public hospitals of tertiary level (the equivalent of a level-1 trauma center) in the region, all of which are references for trauma cases. Besides then, there are also three non-hospital units (equivalent to level-4) and several privet secondary level hospitals (levels 2 and 3). All patients assessed with severe trauma are transferred to level-1 hospitals. This triage is performed at the scene by first responders (paramedics of the FD, or SAMU/GRAU physicians). The destination is defined by a central dispatch, considering the distance from the scene to the hospital, resources at disposal, and hospital capacity at the time. The use of the helicopter limits the options of destinations since only two hospitals in the area have the necessary infrastructure to receive the aircraft, both level-1. This is an important characteristic since air rescue can overload these services with patients who, in some cases, could have been assisted in level 2–3 centers, sometimes closer to the accident site. Other times, it generates interfacility ground transport, which can raise the costs related to that rescue.

In this context, we performed an observational and retrospective study to evaluate the hospital records and prehospital response team records of individuals aged <18 yo, victims of trauma, assisted by GRAU's helicopter rescue team, and transferred to a tertiary university hospital between the years of 2010 and 2018.

We collected the following patients' features using the medical records: (i) demographic data (sex and age); (ii) mechanism of trauma (such as vehicular collisions, run-overs, fall, drowning, burning, gunshot, and knife wounds); and (iii) description of anatomical injuries to calculate the Injury Severity Score (ISS). In addition, we assessed the outcomes of in-hospital death, need and length of stay in the Intensive Care Unit (ICU), complications (nosocomial pneumonia, sepsis, wound infection, and acute respiratory distress syndrome - ARDS), and total length of hospital stay (LOS). Finally, using GRAU prehospital medical records, we collected vital signs and anatomical injuries data to calculate the Revised Trauma Score (RTS) and Pediatric Trauma Score (PTS).

We calculated the ISS after admission based on the description of anatomical injuries evidenced during hospitalization, found through clinical evaluation, image exams, during surgery, or after necropsy. The score is based on the Abbreviated Injury Scale (AIS), which lists several types of injuries found in different body segments, scoring their severity from 1 to 6 (1 being “mild” and 6 “almost always fatal”). ISS, in turn, is calculated by the sum of the squares of the three highest AIS scores, then finally classified as (mild) 1 to 8 points; (moderate) 9 to 15 points; (severe) 16 to 24 points; (very severe) 25 to 75 points. Besides its use for severity classification, it is also used to calculate overtriage, and in our study, we followed two distinct definitions found in the literature: ISS <9 or ISS <16 (6, 10, 13–15).

PTS is used in prehospital assessment and is given by the sum of scores based on patient weight, airway patency, systolic blood pressure (SBP), level of consciousness, external injuries, and bone fractures, each receiving a value of −1, +1, or +2. The PTS can range from −6 to 12, and this trauma score is associated with higher mortality risk when PTS ≤8. RTS can be used for prehospital or in-hospital assessment and is calculated based on SBP, Glasgow coma scale (GCS), and respiratory rate (RR). Prehospital RTS ranges from 0 to 12, and the lowest the score, the most severe is the trauma. Reference to a trauma center is recommended when RTS ≤11. Both scores were analyzed isolated and combined with ISS to correlate the presence of overtriage and undertriage with severity classification at the scene and to analyze the accuracy of these scores having ISS as a standard. These were defined as follows:

- Overtriage described in patients with severe trauma according to PTS or RTS at the scene: those with (PTS ≤8 and ISS ≤15) or (RTS ≤11 and ISS ≤15), respectively.
- Undertriage described in patients with non-severe trauma according to PTS or RTS at the scene: those with (PTS >8 and ISS >15) or (RTS >11 and ISS >15), respectively.

We did the statistical analysis using the descriptive approach with absolute and relative frequencies for categorical data; and the presentation of median (IQR, interquartile range) for numerical data. We also compared the patients according to the outcomes and overtriage/undertriage classified as described before using the Fisher's Exact test, and we adopted an alpha error of 0.05. The statistical analysis was done using the IBM SPSS Statistics for Macintosh, Version 27.0. The Ethics Committee of the institution approved the study (No. 2,739,302).

## Results

We included 49 patients, 39 (79.6%) of whom were male individuals. The mean age was 11 ± 5.6 yo, and the majority were older than 15 yo (17; 34.7%). Automobile accident was the most frequent mechanism of trauma, mainly due to vehicle collisions (15; 30.6%) followed by run-overs (13; 26.5%). Regarding the place of injury, head trauma was predominant (28; 57.1%), followed by severe trauma of lower limbs (15; 30.6%) and thoracic trauma (15; 30.6%); in addition, we also described abdominal trauma (10; 20.4%), pelvic trauma (5; 10.2%), and spinal cord injury (SCI; 4; 8.2%) (Table 1).

**Table 1:** Description of the demographics, mechanism and location of the trauma, secondary outcomes, and severity according to the Injury Severity Score (ISS) and prehospital triage scores.

**Category**	**Distribution**
**Sex**	
Male	39/49 (79.6%)
**Age (years)**	
≤1	2/49 (4.0%)
>1 and ≤5	9/49 (18.4%)
>5 and ≤10	9/49 (18.4%)
>10 and ≤15	12/49 (24.5%)
>15 and ≤18	17/49 (34.7%)
**Mechanism of trauma**	
Vehicular collisions	15/49 (30.6%)
Run-overs	13/49 (26.5%)
Fall	4/49 (8.2%)
Drowning	6/49 (12.2%)
Gunshot wounds	1/49 (2.0%)
Others	10/49 (20.4%)
**Location of trauma**	
Head trauma	28/49 (57.1%)
Severe lower limbs trauma	15/49 (30.6%)
Thoracic trauma	15/49 (30.6%)
Abdominal trauma	10/49 (20.4%)
Pelvic trauma	5/49 (10.2%)
Spinal cord injury	4/49 (8.2%)
**Length of stay (days)**	11 (5 to 22)
**Intensive care unit (ICU) admission**	
Yes	34/49 (69.4%)
ICU length of stay (days)	7 (3 to 14)
**ISS**	
1 to 8 (mild)	9/49 (18.4%)
9 to 15 (moderate)	11/49 (22.4%)
16 to 24 (severe)	1/49 (2.0%)
25 to 75 (very severe)	28/49 (57.1%)
***Revised Trauma Score*** **(RTS)**	10 (8 to 12)
RTS ≤11	18/25 (72.0%)
RTS >11	7/25 (28.0%)
Invalid records^†^	24/49 (49.0%)
RTS ≤11 and ISS ≤15	6/18 (33.3%)
RTS >11 and ISS >15	3/7 (42.8%)
***Pediatric Trauma Score*** **(PTS)**	5 (3–5.5)
PTS ≤8	29/49 (59.2%)
PTS >8	3/49 (6.1%)
Invalid records^††^	17/49 (34.7%)
PTS ≤8 and ISS ≤15	7/29 (24.1%)
PTS >8 and ISS >15	0/3 (0.0%)

*We presented the data using n/N (%) or median (IQR, interquartile range)*.

† *Incomplete data in the prehospital records making it impossible to calculate RTS*.

†† *Incomplete data in the prehospital records making it impossible to calculate PTS*.

**Figure 1:**
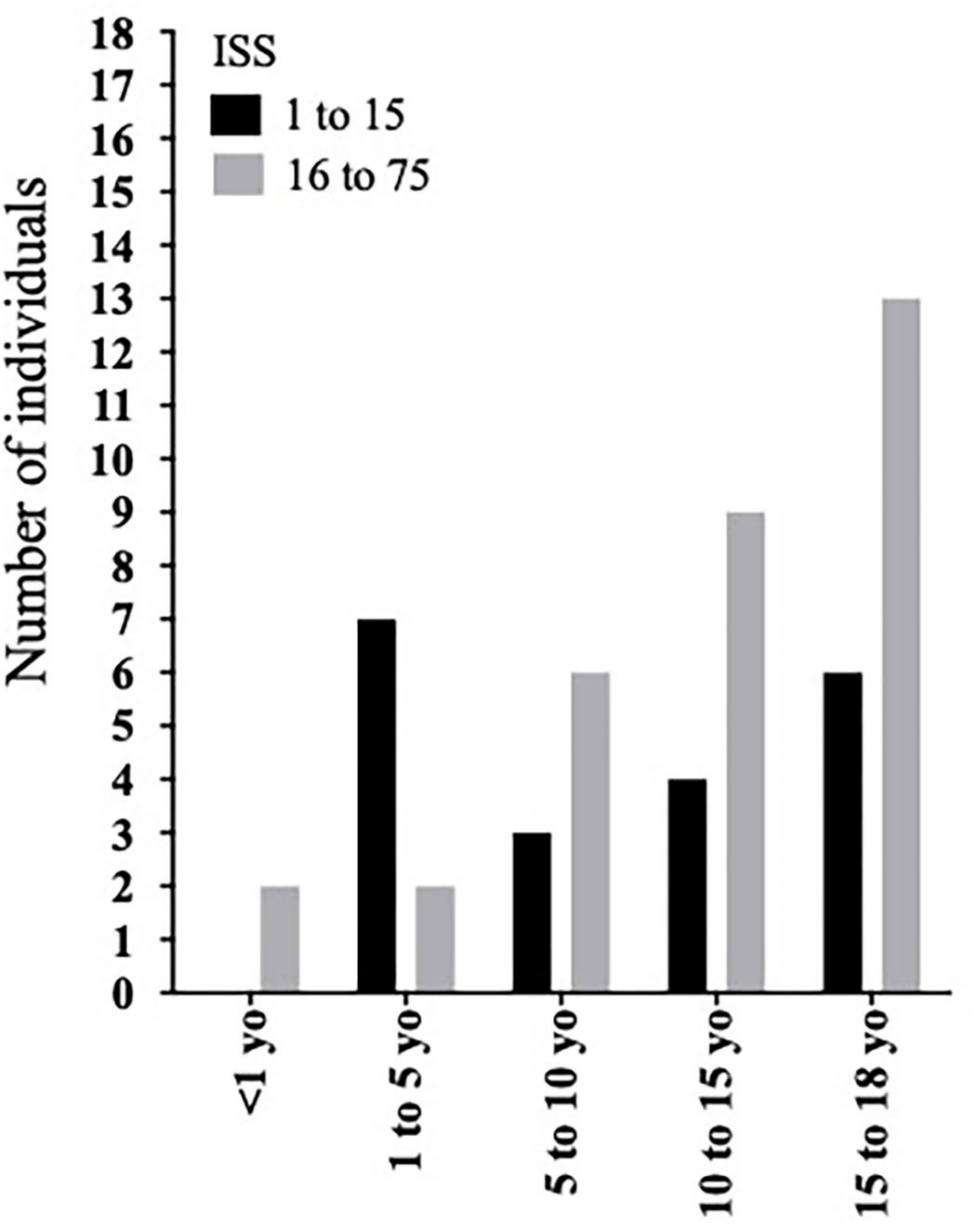
Overtriage distribution in different age groups according to ISS <16 as a cutoff.

**Figure 2:**
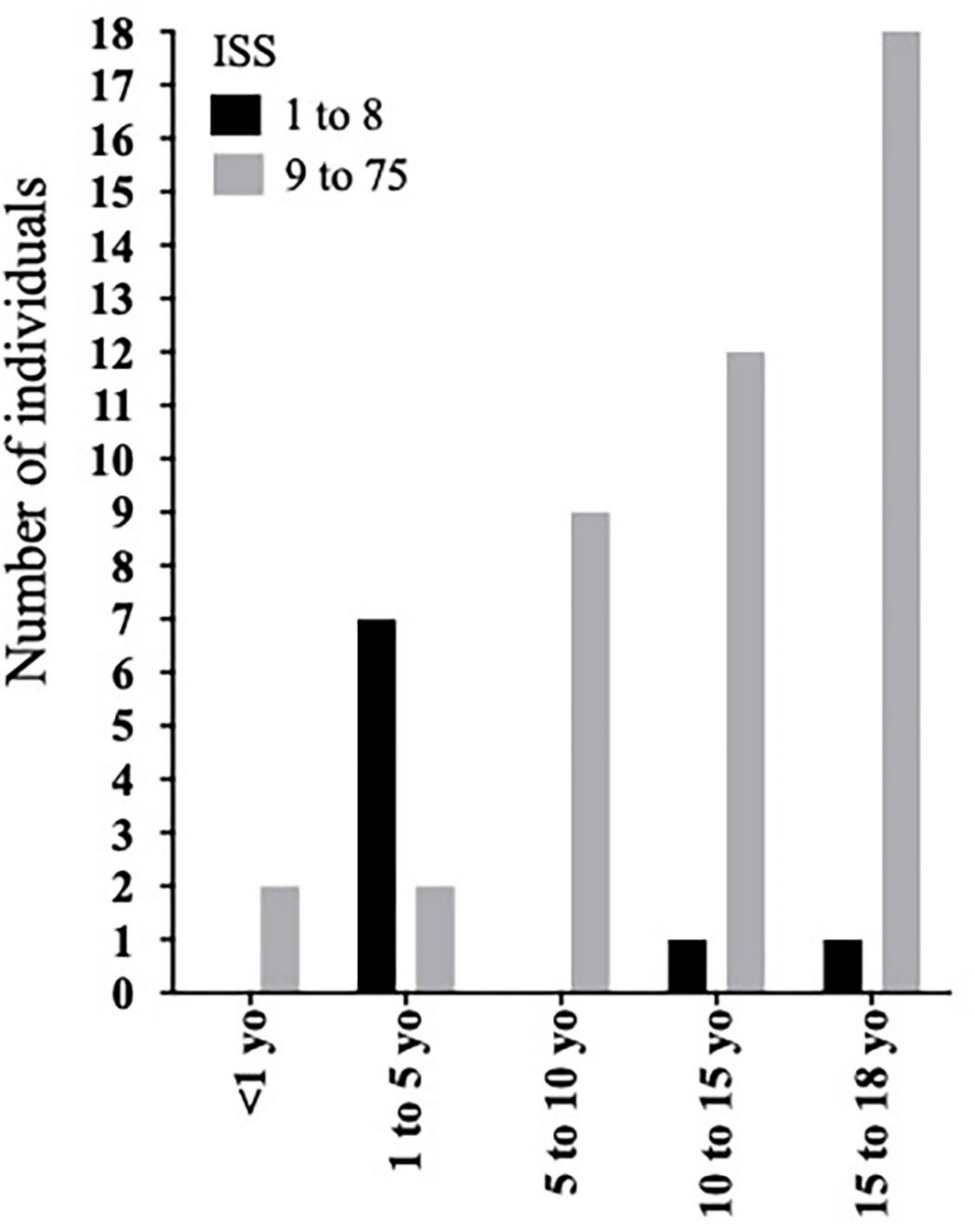
Overtriage distribution in different age groups according to ISS <9 as a cutoff.

ISS was classified as follows: (mild) 9 (18.4%) individuals; (moderate) 11 (22.4%) individuals; (severe) 1 (2%) individual; and (very severe) 28 (57.1%) individuals (Table 1). When analyzing ISS according to age group, we found a predominance of mild and moderate trauma (ISS <9 and <16, respectively) in patients aged between 1 and 5 yo (Figures 1, 2). Finally, a significantly higher chance of survival was associated with ISS <16 (Table 2).

**Table 2:** Distribution of overtriage and undertriage according to their different definitions and mortality in each group.

**Category**	**Death (*n*; %)**	**Survival (*n*; %)**	***p*-value**
**Overtriage**			
ISS <9	0/9 (0%)	9/9 (100.0%)	>0.05
ISS <16	1/20 (5%)	19/20 (95.0%)	0.034
ISS <9 and no need of ICU	0/5 (0%)	5/5 (100.0%)	>0.05
PTS ≤8 and ISS ≤15	0/7 (0%)	7/7 (100.0%)	>0.05
RTS ≤11 and ISS ≤15	1/6 (16.7%)	5/6 (83.3%)	>0.05
**Undertriage**			
PTS >8 and ISS >15	0/0 (0%)	0/0 (0.0%)	>0.05
RTS >11 and ISS >15	0/3 (0%)	3/3 (100.0%)	>0.05

*We presented the data using n/N (%). We did the statistical analysis using the Exact Fisher test, and we adopted an alpha error of 0.05*.

*ISS, Injury Severity Score; ICU, intensive care unit; PTS, Pediatric Trauma Score; RTS, Revised Trauma Score*.

The PTS was 5 (3–5) points, and 29/32 individuals (90.6% of valid cases) presented a PTS ≤8. Of those, 7/29 (24.1%) had an ISS ≤15, indicative of overtriage. Moreover, 3/32 (9.4% of valid cases) presented PTS >8, none of which with ISS >15. The RTS was 10 (8–12) points, and 18/25 individuals (70.2% of valid cases) had the RTS ≤11, from which 6/18 (33.3%) also had ISS ≤15. In addition, we described 7/25 (28% of valid cases) patients with RTS >11. From those, 3/7 (42.8%) patients presented ISS >15, which could be characterized as undertriage at the scene if the patients were not transferred to a trauma center (Table 1).

The average LOS was 11 (5–22) days, and 34 (69.4%) of individuals required admission to the ICU with a length of stay of 7 (3–14) days (Table 1). We also assessed the percentage of four complications during hospitalization, 5 (10.2%) individuals had nosocomial pneumonia, 4 (8.2%) developed sepsis, 4 (8.2%) wound infection, and none had ARDS.

The death occurred in 10/49 (20.4%) individuals on 4 (0.75–6.75) days after the accident. There was no significant difference in the number of deaths according to sex, age, severity, or trauma mechanism. But a higher percentage of deaths was described associated with thoracic trauma (*p* = 0.049) and SCI (*p* = 0.017) (Table 3).

**Table 3:** Distribution and description of mortality according to demographic data, severity, and mechanism and location of trauma.

**Category**	**Distribution**
Mortality (prevalence)	10/49 (20.4%)
Time of death from admission (days)	4 (0.75 to 6.75)
**Sex**	
Masculine	7/39 (17.9%)
Feminine	3/10 (30.0%)
**Age (years)**	
≤1	1/2 (50.0%)
>1 and ≤5	0/9 (0.0%)
>5 and ≤10	2/9 (22.2%)
>10 and ≤15	1/12 (8.3%)
>15 and ≤18	6/17 (35.3%)
**Mortality according to ISS**	
0–8	0/9 (0.0%)
9–15	1/11 (9.1%)
16–24	0/1 (0.0%)
25–75	9/28 (32.1%)
**Mortality rate according to the PTS**	
≤8	8/29 (27.6%)
>8	1/3 (33.3%)
**Mortality rate according to the RTS**	
≤11	8/18 (44.4%)
>11	0/7 (0.0%)
**Mortality rate according to the mechanism of trauma**	
Vehicular collisions	2/15 (13.3%)
Run-overs	2/13 (15.4%)
Fall	2/4 (50.0%)
Drowning	1/6 (16.7%)
Gunshot wounds	0/1 (0.0%)
Others	3/10 (30.0%)
**Mortality according to the location of trauma**
Head trauma	7/28 (25.0%)
Severe lower limbs trauma	2/15 (13.3%)
Thoracic trauma	6/15 (40.0%)
Abdominal trauma	3/10 (30.0%)
Pelvic trauma	2/5 (40.0%)
Spinal cord injury	3/4 (75.0%)

*We presented the data using n/N (%) or median (IQR, interquartile range)*.

*ISS, Injury Severity Score; RTS, Revised Trauma Score; PTS, Pediatric Trauma Score*.

## Discussion

Helicopter rescue in metropolitan areas can be useful due to the great distances to be traveled, heavy traffic, and difficulties in accessing specific locations; such factors could compromise response time, negatively affecting the outcomes of trauma cases. However, the study of its benefits showed conflicting results. While some studies fail to demonstrate any difference between air and ground transport of the trauma victim (whether in mortality, the need for ICU, or LOS) (13, 14), others demonstrate benefits but have difficulties in defining which elements are responsible for such results (14–17).

Brow *et al*. (5) comparing air vs. ground rescues in children up to 16 years, in a five-year period, described an improvement in immediate survival in the individuals assisted by the helicopter, but with no difference in deaths occurring after 48 h (hs). Also, Englum *et al*. (6) described a lower mortality rate due to air rescue only in moderate and severe trauma. Another study by Brown *et al*. evaluating survival concerning the time of transportation from scene to hospital, found benefit only when this time frame was between 6 and 30 min (14). The literature described that reduction in transport time alone does not explain the phenomenon (9, 12) and that the time spent at the scene is often even greater (4, 14, 18). Thus, when demonstrated in specific subpopulations, the mechanism of this benefit is probably due to multiple causes (19).

The lack of solid evidence about the helicopter's real benefit and which individuals it would benefit the most hinders the establishment of clear protocols. In addition, higher operational costs and safety concerns (1, 2, 5, 11) make the improvement in the management of air rescue dispatch even more critical.

In our data, the epidemiological profiles of pediatric patients assisted by helicopter rescue demonstrated a high prevalence of male adolescents, mainly victims of car accidents. Sex, mean age, and the number of cases of children aged 10 years and over followed the literature, as did the prevalence of blunt trauma, with a higher frequency of vehicular collisions and run-overs (4, 5, 9, 10, 20).

Most of the indications of air rescue support found in the Military Police Operational Protocols (MP-POPs) are frequently found in road accidents scenarios. For example, long-distance between the incident and hospital, no advanced ground support team nearby, critical traffic and assistance to multiple victims, and this may have contributed to the high frequency of car related trauma mechanisms in our cohort. Our study did not assessed the specific use of aeromedical support according to military police POPs. Cardoso *et al*. evaluating the same service from 2010 to 2012, demonstrated that approximately 26% of the patients rescued by helicopter in the area did not fit any of the criteria found in the protocols and concluded the need for improvement in the triage system (7).

Our analysis focused on the indications based on anatomical injuries and physiological changes. Unlike what has been described in international studies (4–6, 10, 21), most children who had severe trauma in our data presented an ISS ≥25. Our finding is significant since ISS is the main score used to characterize overtriage in trauma in the literature. However, some authors also associate the overtriage criteria with the need for early critical resources (such as ICU admission, non-orthopedic surgery, or transfusion) not relying on the ISS classification alone (21, 22). Defined as the screening decision that results in unnecessary use of resources or personnel (10), overtriage is common in aeromedical rescues (6) since different protocols and perceptions of severity by the crew can lead to subjectivity in the victims' assessment. In addition, first responders must also take care in avoiding undertriage, underestimating the urgency of the patient's condition, and perhaps erroneously saving resources or not transferring the patient to a trauma center, leading to a significant increase in mortality (7, 21).

The American College of Surgeons considers up to 5% of undertriage in traumatized patients acceptable, which results in 25 to 35% of overtriage. These are considered the targets by most specialized committees (8). According to the criteria used for its classification, overtriage varies from 30 to 70% in air rescues, reaching up to 90% being even more prevalent in the pediatric population (3–5, 9, 13). A meta-analysis by Bledsoe et al. demonstrated 60% of overtriage according to an ISS <16 (23) while Knofsky *et al*. and Brown *et al*. reported this index around 70% (5, 20) and Eckstein et al. 83% (24). The lowest rates of overtriage based on this ISS cutoff were found in Steward *et al*. (55.4%) and Mulholland *et al*. (46.4%) (4, 9). In our sample, 40.8% of the cases presented an ISS <16. Still above the acceptable consensus, but less than what was found in those studies. The same occurred with ISS <9 as a cutoff for overtriage, a definition chosen by other authors, such as Englum *et al*. and Michailidou et al., who reported 38 and 41.1%, respectively, compared to 18.4% in our data (6, 10).

In our cohort, we attributed the predominance of overtriage in patients aged between 1 and 5 yo to the mechanism of trauma most prevalent in this age group. Curiously, five of these patients (55.5%) were drowning victims, a mechanism of trauma that does not cause any anatomical injury and, therefore, does not score on the ISS, underestimating the severity based on this index.

Regardless of the value chosen for the definition of overtriage, ISS is a helpful index for classifying the severity of trauma. However, it has the disadvantage of being calculated based on diagnoses during hospitalization, thus not applicable for screening at the scene. Prehospital assessment of severity in pediatric trauma cases can be challenging due to the child's anatomy and physiology (21, 25, 26). The sensitivity of pediatric prehospital triage tools ranging has been reported to range from 49.1% to 87.3% (21), sometimes resulting in overtriage rates higher than 20% (22).

PTS is a useful tool for pediatric triage trauma and is simple to calculate. This index mixes physiological and anatomical criteria associated with pediatric trauma's major causes of death. One of its disadvantages is the greater emphasis on external injuries and the subjectivity in its classification and airway patency. Studies showed a good correlation between PTS and ISS in the identification of severe trauma (ISS >15) (27, 28) and consider eight as the critical cutoff. Ramenofsky *et al*. were the first to define this value, reporting the absence of deaths when PTS >8, increasing to 24% when ≤8 (29). Similarly, Kaufmann *et al*. and Eichelberger *et al*. reported 13 and 30% mortality, respectively, when PTS ≤8 and no deaths above that value (27, 28). In our data, only 1 out of 10 deaths occurred with a PTS >8. This individual was a victim of SCI, after a shallow water dive, and presented a PTS of 10 and RTS of 12 in prehospital triage and a final ISS of 9. The death was not due to the primary trauma but 40 days after the accident, secondary to sepsis (pulmonary focus).

In analyzing the accuracy of PTS as a screening tool, we used the definition of overtriage as PTS ≤8 (high mortality) with ISS ≤15 (non-severe), and we found 24.1% of cases in our data. At the same time, the studies mentioned above described rates from 21.5 to 42.6% (27, 28). Among those individuals with PTS >8, no ISS >15 (severe trauma) was described, thus showing no undertriage at the scene according to this score (28).

In addition to anatomical injuries, physiological changes are suitable parameters for screening at the scene because they are objective and easy to assess. RTS is another widely used index, but in pediatrics, it has the disadvantage of not being corrected for age, on which the normal values of SBP and RR depend. Most authors recognize RTS ≤11 as an indicator of severity and the need for transportation to a trauma center (27, 28, 30). In our cohort, 28% of valid RTS were above 11, and we found 33.3% of RTS overtriage at the scene (defined as RTS ≤11 and ISS ≤15), like the 19.5 to 32% range described in the literature (27, 28). However, we found 42.8% undertriage at the scene when analyzing the individuals with RTS equal to 12 and ISS >15. This result was well above what is acceptable and described in international studies (8, 21, 27, 28). It may have been influenced by missing data from prehospital records, reducing the number of valid samples for analysis.

Regarding patients' outcomes, LOS had a median of 11 days in our data, ranging from 3 to 8 days in the surveyed literature (5, 6, 9). Discharge in <24 h is another criterion commonly used to characterize overtriage, and it is described in 25% to 30% of children's' air rescues (5, 10, 20). Although in our cohort three patients were discharged after observation only (on average, 28 h after the trauma), there was no discharge case in the first 24 h of admission.

The prevalence of ICU admission was 69.4%. In contrast, Mulholland et al. and Brown et al. described a prevalence of around 40% while Michailidou et al. reported 33% (5, 10, 11). Following this trend, mortality in our study was 20.4%, while it varied from 4 to 7.5% in the literature reviewed (4–6, 9). Of these deaths, 90% occurred in individuals with ISS ≥25, and one death occurred in one individual with ISS equal to 9 due to SCI, as previously described. When analyzing overtriage in the context of the need for ICU, it is essential to note that some authors exclude patients from the overtriage classification, who, despite having an ISS <9 or <16, were admitted to the ICU (21). In our sample, this corresponds to 44.4% of the patients with ISS <9 and 45% of those with ISS <16. This percentage would fall to 0 and 25%, respectively, with the exclusion from this analysis of four drowning victims (all ISS = 1).

The mortality of our sample was mainly associated with primary injuries, occurring on median of 4 days after the trauma. The percentage of severe complications was small. In addition, the predominance of wound infections in severe lower limb injuries can be associated with the presence of external fixators, frequently used in these cases.

The authors acknowledge that the present study has some limitations. First, the incomplete filling of some hospital medical records and prehospital records resulted in the absence of some crucial data for calculating trauma scores at the scene (mainly RTS). The second is the low number of patients. But in this regard, although it may at first seem small, we consider it a reflection of the small contingent of pediatric trauma in the region, which has been decreasing in the last decade, especially with the severity and other characteristics necessary to the indication of air rescue.

## Conclusions

Helicopter transport of pediatric trauma in the region during the study period was used mainly for critically ill patients. This resulted in overtriage rates that, although above ideal, are compatible (or lower, depending on the criteria adopted) with those described by international literature, and in more consolidated and longer established services. Of the severity screening tools used at the scene, PTS showed the lowest rate of overtriage within an excellent undertriage rate. Further studies are necessary, evaluating the accuracy of pediatric screening at the scene and comparing the benefits of helicopter vs. ground transport in Brazil.

## Data Availability Statement

The original contributions presented in the study are included in the article/Supplementary Material, further inquiries can be directed to the corresponding author/s.

## Ethics Statement

The studies involving human participants were reviewed and approved by Ethics in Research Committee - Faculty of Medical Sciences, University of Campinas (Protocol # 2,739,302). Written informed consent from the participants' legal guardian/next of kin was not required to participate in this study in accordance with the national legislation and the institutional requirements.

## Author Contributions

All authors listed have made a substantial, direct, and intellectual contribution to the work and approved it for publication.

## Conflict of Interest

The authors declare that the research was conducted in the absence of any commercial or financial relationships that could be construed as a potential conflict of interest.

## Publisher's Note

All claims expressed in this article are solely those of the authors and do not necessarily represent those of their affiliated organizations, or those of the publisher, the editors and the reviewers. Any product that may be evaluated in this article, or claim that may be made by its manufacturer, is not guaranteed or endorsed by the publisher.
