# Supplementary material for: Air Rescue for Pediatric Trauma in a Metropolitan Region of Brazil: Profiles, Outcomes, and Overtriage Rates
Source: Front Pediatr. 2022 Jun 2;10:890405. doi: 10.3389/fped.2022.890405 (PMC9201391; doi:10.3389/fped.2022.890405)
Supplement: Supplementary file 1 [file Data_Sheet_1.PDF]

Supplement: Epidemiologic profile of the individuals rescued by helicopter due to Pediatric Trauma in the Metropolitan Region of Campinas, from 2010 to 2018

**Individual**	Age (yr)	Sex*	Trauma mechanism	PTS	RTS	ISS	Death**	ICU LOS†	Complications	LOS†	Trauma location††
**1**	0.3	F	Vehicular Collision			25	Yes, less than 1 day	<1			TBI
**2**	13.8	F	Fall	-3	2	50	Yes; 1 day	1			TBI, thoracic, abdominal, pelvic, lower limbs and SCI
**3**	16.3	M	Vehicular Collision	-3		75	Yes; at admission				Thoracic and lower limbs
**4**	6.9	M	Run over	-2	0	50	Yes; 3 days	3			TBI, thoracic, abdominal and SCI
**5**	10.1	F	Run over	0	0	38	Yes; 4 days				TBI, thoracic, abdominal, and pelvic
**6**	16.2	M	Fall	3	9	75	Yes; 4 days				TBI and thoracic
**7**	17.3	M	Drowning	3	0	75	Yes; 5 days				
**8**	16.8	M	Other	3	10	75	Yes; 12 days		Pneumonia; sepsis		TBI
**9**	15.3	M	Other	5	10	34	Yes; 5 days				TBI and thoracic
**10**	17.3	M	Other	10	11	9	Yes; 41 days	29 plus 12	Pneumonia; sepsis		SCI
**11**	4.8	M	Vehicular Collision			2				1	
**12**	11.6	M	Run over			8				1	TBI
**13**	15.7	M	Vehicular Collision	11	12	14				1	TBI
**14**	1.3	M	Drowning			0		2		2	
**15**	4.6	F	Drowning			0				2	
**16**	16.3	M	Other	5	10	26				3	TBI
**17**	15.8	M	Vehicular Collision			9				5	Lower limbs
**18**	0.6	M	Vehicular Collision	2		26		5		5	TBI
**19**	17.4	M	Gunshot wound	3		5				5	
**20**	1.3	M	Run over	5	12	57		1		5	TBI, thoracic and abdominal
**21**	14.4	M	Vehicular Collision	8	12	9				5	Lower limbs
**22**	2.8	M	Drowning			0		3		6	
**23**	11.2	M	Other			26		2		8	TBI and abdominal
**24**	2.9	M	Fall			29				8	TBI
**25**	3.4	M	Drowning	2	0	0		6		8	
**26**	3.3	M	Other			4			Wound infection	9	Lower limbs
**27**	13.8	M	Run over	6	11	10		2		9	Lower limbs
**28**	7.6	M	Run over	4		34		3		10	TBI and lower limbs
**29**	4.8	M	Drowning	8	10	0		8		10	
**30**	6.1	M	Run over	4	8	9		3	Wound infection	11	TBI
**31**	5.3	F	Vehicular Collision	10	12	9				12	Pelvic
**32**	11.3	M	Run over			38		7		13	TBI and abdominal
**33**	15.4	F	Vehicular Collision	4	11	57		7		13	TBI, thoracic, abdominal, lower limbs and SCI
**34**	9.9	M	Run over		9	10		8		15	TBI
**35**	14.8	M	Other	5		26		6		15	TBI
**36**	17.8	M	Vehicular Collision	8	12	10				16	Lower limbs
**37**	15.0	M	Run over	4	10	38		14	Pneumonia	17	TBI and thoracic
**38**	12.6	F	Vehicular Collision	5	8	34		5		20	TBI and thoracic
**39**	14.7	F	Vehicular Collision			27		16		22	TBI, thoracic and abdominal

**40**	16.1	M	Other			9				22	Lower limbs
**41**	17.7	M	Run over	3		26		17	Pneumonia	24	TBI
**42**	6.4	M	Fall	4	12	41		8		28	Thoracic, abdominal and pelvic
**43**	14.3	F	Vehicular Collision			32		12		32	TBI and thoracic
**44**	15.5	M	Vehicular Collision			13		2		37	Lower limbs
**45**	14.6	M	Running over	2		43		38	Sepsis	38	TBI, thoracic and lower limbs
**46**	16.2	M	Other			20		6		42	Lower limbs
**47**	5.7	F	Other	4		25		30		46	TBI
**48**	17.3	M	Vehicular Collision	4	10	34		17	Wound infection; sepsis; pneumonia	56	TBI and lower limbs
**49**	10.4	M	Run over	6	12	27		9	Wound infection	74	Thoracic, abdominal, lower limbs and pelvic

* M, male; F, female
** When death present, time frame of death was given in days after admission
† ICU LOS, Intensive Care Unit length of stay (days); LOS, total hospital length of stay (days)
†† TBI, traumatic brain injury; SCI, spinal cord injury
ISS, Injury Severity Score; RTS, Revised Trauma Score; PTS, Pediatric Trauma Score
