# Supplementary material for: Air Rescue for Pediatric Trauma in a Metropolitan Region of Brazil: Profiles, Outcomes, and Overtriage Rates
Source: Front Pediatr. 2022 Jun 2;10:890405. doi: 10.3389/fped.2022.890405 (PMC9201391; doi:10.3389/fped.2022.890405)
Supplement: Supplementary file 2 [file Data_Sheet_2.PDF]

Supplement: Trauma scores for severity classification

Pediatric Trauma Score (PTS)

PTS is used in prehospital assessment and is given by the sum of scores based on five variables (described in the table bellow), each receiving a value of -1, +1, or +2. The score can range from -6 to 12, and it is associated with higher mortality risk when ≤8

**Score**	**+2**	**+1**	**-1**
**Weight**	> 20 kg	10 - 20 kg	< 10 kg
**Airway**	Normal	Maintainable	Unmaintainable
**Systolic Blood Pressure**	> 90 mmHg	50 - 90 mmHg	< 50 mmHg
**Level of Consciousness**	Awake	Obtunded, loss of consciousness	Comatose, unresponsive
**Bone fractures**	None	Single, closed	Open or Multiple
**External injuries**	None	Small and closed wounds	Large, penetrating, burns

Revised Trauma Score (RTS) – Prehospital version

RTS can be used for prehospital or in-hospital assessment and is calculated based on three variables scored as described in the table bellow. Prehospital RTS is calculated by the simple sum of each score. It ranges from 0 to 12, and the lowest the score, the most severe is the trauma. Reference to a trauma center is recommended when RTS ≤11.

Glasgow Coma Scale	Value	Systolic Blood Pressure (mmHg)	Value	Respiratory Rate	Value
13-15	4	>89	4	10-29	4
9-12	3	76-89	3	>29	3
6-8	2	50-75	2	6-9	2
4-5	1	1-49	1	1-5	1
3	0	0	0	0	0

Injury Severity Score (ISS)

ISS is based on the Abbreviated Injury Scale (AIS), which lists several types of injuries found in different body segments, scoring their severity from 1 to 6, as follows: 1 (Minor), 2 (Moderate), 3 (Serious), 4 (Severe), 5 (Critical) and 6 (Maximum, currently untreatable, almost always fatal).

ISS assess six body regions (not coinciding with the ones described in the AIS 2005 - Update 2008 dictionary): (i) head and neck, (ii) face, (iii) chest, (iv) abdomen, (v) extremity, including pelvis and (vi) external.

After scoring each region according to the AIS, ISS is then calculated by the sum of the squares of the three highest AIS scores, and finally classified as (mild) 1 to 8 points; (moderate) 9 to 15 points; (severe) 16 to 24 points; (very severe) 25 to 75 points.

A demonstration can be seen in the following example:

**ISS Body Region**	**Injury**	**AIS score**	**AIS^2**
Head and neck	Skull base fracture without CSF leak	3	
Face	Maxillary fracture	3	9
Chest	Unilateral pneumothorax	3	9
Abdomen	Spleen rupture	4	16
Extremity/Pelvis	Closed pelvic fracture	2	
External	Superficial abrasions	1	
**ISS**			**34 (very severe)**
